# Female Volleyball Players Are More Prone to Cortisol Anticipatory Stress Response than Sedentary Women

**DOI:** 10.3390/medicina55060258

**Published:** 2019-06-08

**Authors:** Inga Dziembowska, Małgorzata Wójcik, Iga Hołyńska-Iwan, Kamila Litwic-Kaminska, Artur Słomka, Ewa Żekanowska

**Affiliations:** 1 Department of Pathophysiology, Faculty of Pharmacy, Ludwik Rydygier Collegium Medicum in Bydgoszcz, Nicolaus Copernicus University in Toruń, Skłodowskiej-Curie 9, 85-094 Bydgoszcz, Poland; artur.slomka@cm.umk.pl (A.S.); zhemostazy@cm.umk.pl (E.Ż.); 2 Department of Physiotherapy, Stanisław Staszic University of Applied Science in Piła, Podchorążych 10, 64-920 Piła, Poland; malgo_wojcik@interia.pl; 3 Department of Pathobiochemistry and Clinical Chemistry, Faculty of Pharmacy, Ludwik Rydygier Collegium Medicum in Bydgoszcz, Nicolaus Copernicus University in Toruń, Skłodowskiej-Curie 9, 85-094 Bydgoszcz, Poland; igaholynska@cm.umk.pl; 4 Institute of Psychology, Kazimierz Wielki University in Bydgoszcz, Staffa 1, 85-867 Bydgoszcz, Poland; k.litwic@ukw.edu.pl

**Keywords:** salivary cortisol, heart rate, female athletes, anxiety, mental stress

## Abstract

*Background and Objectives:* Top-level sports performance places heavy physical and psychological demands on elite-level athletes, which can be a source of increased levels of stress. Therefore, high-level volleyball players may present altered cardiovascular and endocrinological stress response during stressful events. Although many previous studies have examined the response to stress on athletes, most of them regarded only males, while the impact of the female menstrual cycle has rarely been taken into account. We aimed to study psychophysiological response to anticipatory stressor through analysis of heart rate, self-reported anxiety level, and salivary cortisol in healthy young female athletes by minimalizing the effect of confounders. *Materials and Methods:* A total of 55 females (25 members of the best league for female volleyball players in Poland and 30 sedentary-lifestyle control subjects) in the follicular phase of their menstrual cycle were exposed to mental arithmetic tasks as an experimental imitation of the stressor. Volleyball players were significantly taller than sedentary individuals (177.1 ± 3.4 cm vs. 173.3 ± 3.4 cm, respectively, *p* = 0.034), but did not differ in weight (73.6 ± 5.2 kg vs. 70 ± 4.23 kg, respectively, *p* = 0.081), body mass index (BMI) (23.5 ± 1.13 vs. 24.1 ± 1.45, respectively, *p* = 0.060), and age (22 ± 1.11 vs. 23 ± 1.14 years, respectively, *p* = 0.2). Their stress responses were assessed through self-reported anxiety levels and physiological measurements of salivary cortisol concentrations and heart rate (HR). *Results:* For HR, significant effects of time (F_(2,120)_ = 21.34, *p* < 0.001, η^2^ = 0.26) were found, but not for training status (F_(1,60)_ = 2.69, *p* = 0.106, η^2^ = 0.04). For cortisol levels, the analysis showed the main effects of time (F_(3,180)_ = 11.73, *p* < 0.001, η^2^ = 0.16) and training status (F_(1,60)_ = 4.69, *p* = 0.034, η^2^ = 0.07) and a significant interaction between training status and time (F_(3,180)_ = 3.07, *p* = 0.029, η^2^ = 0.05). Post-hoc analyses showed higher cortisol concentrations among volleyball players following the math task (all *p* < 0.001), as well as higher cortisol concentrations in S2, S3, and S4 compared to S1 in volleyball players (all *p* < 0.001). We observed also a significant increase in state anxiety in both groups (all *p* < 0.001), but no differences in state anxiety levels between groups. *Conclusion:* Female volleyball players may not differ in subjective graduation of stressors; however, exposure to training-based stressors seems to promote cortisol response to the anticipated stressor.

## 1. Introduction

Competitions and expectations to perform to a high standard are a regular source of physiological and psychological stress for athletes [1]. A stressful event induces behavioral and biochemical changes including the activation of the hypothalamic–pituitary–adrenal (HPA) axis and the sympathomedullary (SAM) pathway [2]. These changes are an adaptive mechanism that enables the body to maintain homeostasis in conditions of increased mobilization. Activation of the SAM system leads to epinephrine secretion. Epinephrine influences cardiovascular activity, leading to an increase in heart rate (HR), blood pressure (BP) as well as metabolic changes, such as an increase in the oxidative stress [3]. Activation of SAM also results in the secretion of salivary alpha-amylase, which appears to be a good candidate for a biochemical marker of SAM activation [4,5].

Subsequently, the hypothalamic–pituitary–adrenal (HPA) axis activation elicits a substantial increase in cortisol levels [6]. Cortisol is known to affect the brain and body functions associated with mobilization of energy sources and stress management [7,8,9]. Physiologically, cortisol levels produce a circadian rhythm, with the largest release in the morning, 20–45 min after awakening (which is known as cortisol awakening response or CAR), and the lowest concentrations following the first few hours of sleep.

After exposure to a stressor, cortisol blood concentration increases, reaching a maximum between 10 and 30 min, followed by a decrease [7,8,9]. However, every imbalance, such as monthly hormonal fluctuations, pregnancy, and hormonal contraception affect cortisol levels [10,11]. It is noteworthy that only a small amount (less than 5% of total blood cortisol) is present in its unbound, biologically active form [12]. Determination of steroids in serum or plasma will, therefore, present the result determining the total concentration, referring mainly to the inactive hormone, which will provide only an approximate assessment of the hormonal balance but does not allow to assess how much of the active form is contained in the blood in response to stress [5,13]. Unbound cortisol is excreted unchanged in the saliva [14]; therefore, it is much more advantageous to determine only the free fraction of the hormone in the saliva samples. The high correlation between serum cortisol concentrations together with the simplicity of collection makes salivary cortisol, and not serum cortisol, preferable as an HPA axis indicator [5,13].

Previous research has suggested that physical fitness contributes to the adaptation of physiological stress systems, which was called the cross-stress adaptation (CSA) hypothesis [15]. These changes would have a beneficial effect on the body’s response to stressful situations. In particular, there has been a reduction in the cortisol burst in people showing regular physical activity. In their meta-analysis, Mücke et al. emphasize that about half of the studies suggested that higher levels of physical fitness were associated with altered cortisol response to psychosocial stress [16].

Previous studies, presented in the meta-analysis, indicate that a moderate increase in cortisol levels is observed in athletes before sports competition [17]. This process of anticipatory stress response prepares the athlete’s body and mind for competition through the mobilization of energy levels. Current studies suggest that cortisol released in response to stress works in two ways, with greater cortisol demand for mental tasks, and lower for physical tasks. Furthermore, higher cortisol levels seem to be connected negatively with athletes’ physical performance measured through scoring points of the International Association of Athletics Federation, which may lead to the conclusion that high cortisol response is maladaptive regarding sport performance [18].

In their meta-analysis, van Paridon et al. concluded that female athletes do not have a tendency to show a significant anticipatory cortisol response, which was explained by the specificity of the stressor associated with sports competition engaging both physical and physiological capacities [17]. However, previous studies did not consider the menstrual cycle phase which may have affected the results. High levels of estradiol and progesterone during the luteal phase seem to alter cortisol release in response to socio-evaluative stressors by an easy return to initial values after a stressful situation [19]. As the luteal phase is usually longer than the follicular phase, there is a high probability that a woman recruited for examination at a random moment is in the luteal phase, not follicular. Understanding how female athletes respond to an anticipated stressor, even when a real challenge does not exist, would be a step toward building better coaching strategies. 

The aim of the present study was to investigate the impact of top-level sports performance on anticipatory stress response in healthy women by minimalizing the effect of confounders. Separating the reaction to stress prediction and stress response was intended to clarify whether the mere expectation of stress would cause a release of cortisol in female volleyball players. We aimed to study psychophysiological response to an anticipated stressor through the analysis of heart rate, self-reported anxiety level, and salivary cortisol in healthy young female volleyball players compared to sedentary women.

The use of hormonal contraception and irregular menstrual cycles are a frequent occurrence in female athletes. The restrictive exclusion criteria required a large number of athletes of the same discipline, as we were aware that most of them were not eligible for the study. We chose female volleyball players because team sports represent the majority of sports. Additionally, team sports are characterized by the need for good cooperation not only with the coaches but also with the team members. Hence, the number of stressful situations and their significance for the team’s performance are much greater than in non-team sports [20]. It is worthy to note that the major inclusion criterion, which allowed us decide on volleyball instead of strictly aerobic sports (which seem to generate the best adaptation to other stressors), was not the level of physical fitness, but the many different sources of psychosocial stress. To ensure that participants were under frequent pressures related to sports competitions, we decided to include only members of the A (the best league for female volleyball players) teams.

To simulate a mock stressful situation, we performed a short, mental arithmetic task, with however no time pressure or any form of evaluation of the participant’s answer. As previous research indicates, a mathematical task with a low degree of difficulty without time pressure or a component related to social assessment should not trigger a stress response, which therefore ensured the possibility of assessing only the response to “anticipation of a stressful situation” and not the answer to “a stress situation actually occurring”. However, immediately before the math task, participants were informed that they would have to undergo a task in order to examine their stress management skills (in order to enhance the preparation to the stressor) when the math task began.

As we had to consider menstrual cycle phases and minimalize the possibility of any unexpected stressors and physical activity (which caused the need to adjourn the individual’s experimental session), we chose mornings for the experimental sessions to minimalize interruptions of the volleyball players’ training cycles. As we were aware that research has shown that cortisol levels may be still elevated before noon, the experiments began 3 h after awakening to minimalize the effect of cortisol awakening response (CAR) on results.

## 2. Materials and Methods

### 2.1. Participants

Participants were 55 females, namely, 25 volleyball players and 30 sedentary individuals, ranging in age from 18 to 24 (mean age of athletes 22.0 ± 1.11, mean age of sedentary females: 23.0 ± 1.14 years). The group of professional (fully paid) athletes who voluntarily participated in the study consisted of Polish volleyball players who were members of the A teams (the best league for female volleyball players) with at least five years of training experience and who trained at least four times/week. These participants were recruited through meetings organized at sports camps. Non-athletic control subjects were volunteers who led a sedentary lifestyle and were neither members of a sports club nor reported actively training on a regular basis. The sedentary participants were recruited through posters and leaflets in public places. Interested participants were screened through an initial telephone interview to check if they met the eligibility criteria. If eligible, the perspective participant visited the laboratory and was provided with all the information needed to prepare for the experimental session. Participants’ eligibility for the study was verified through a detailed interview and completing the International Physical Activity Questionnaire (IPAQ), which is a validated tool to measure the level of physical activity [20]. If a participant was eligible, a date for the laboratory visit was arranged. All participants had accomplished secondary education and had acquired a general qualification for university entrance. Data were collected between the 5th and 9th day of the menstrual cycle (follicular phase). Exclusion criteria were as follows:

- irregular menstrual periods within the last 24 months or any history of pregnancy,
- presence of chronic disease, particularly cardiovascular disease or illness that involved disturbance of the HPA,
- previous/current mental disorders,
- heavy nicotine use (smoking >5 cigarettes per day, at least three times per week),
- heavy caffeine use (>300 mg/day) or feeling the side effects of caffeine withdrawal,
- using hormonal contraceptives,
- experiencing traumatic life events within the last six months,
- having had surgery under general anesthesia during the past year,
- underweight (body mass index (BMI) <18) or overweight (BMI >25),
- severe vision or hearing problems,
- for the control group only, moderate or high physical activity level measured by IPAQ [20].

If a participant was eligible, a date for the laboratory visit was arranged. The participants were informed that their stress management would be measured through the measurement of psychological and psychophysiological parameters. However, participants were not informed that they were going to undergo any task at that time. They were also instructed on how to prepare for the experimental session as described in Study Design. The study protocol was approved on 18th March 2014 by the Bioethics Committee of the Nicolaus Copernicus University in Toruń operating at the Collegium Medicum in Bydgoszcz, Poland, and all participants gave their informed consent prior to their inclusion in the study (permit No. KB/247/2014).

### 2.2. Heart Rate Measurement

Heart rate was continuously recorded with an emWavePro^®^ device (HeartMath^®^ Inc., Boulder Creek, CA, USA) using a plethysmographic sensor, which is a reliable tool for heart rate measurement in healthy individuals [21]. This sensor was chosen mainly because it is easy to use and comfortable for the participant, so that additional stress connected with the sensor’s placement on the participant’s body was minimalized [21,22]. The sensor records R–R intervals with a sampling frequency of 1000 Hz, providing a time resolution of 1 ms for each R–R interval. After eliminating artifacts, the HR means were computed using Kubios HRV Standard^®^ software (Version 3.1.0, Biomedical Signal Analysis Group, University of Kuopio, Kuopio, Finland). HR was analyzed according to the task force’s recommendation, at intervals of 5 min [21]. Minutes 0–5 were selected as a pre-arithmetic task, minutes 6–10 as mental stress (arithmetic task), and minutes 11–15 as a post-arithmetic task. 

### 2.3. Salivary Cortisol Level Measurement

The participants provided four saliva samples (S1, S2, S3, and S4) using salivettes (Sarstedt, Nümbrecht, Germany) to measure cortisol levels during the session. The timing for the saliva sampling was performed at pre-task condition (S1) 5 min before the onset of the math task, and then at intervals of 15 min until the end of the experimental session (S2, S3, and S4). Samples were centrifuged at 3000 rpm for 5 min, resulting in a clear supernatant of low viscosity which was frozen at −80 °C until the analysis took place. The samples were analyzed using an Eagle Biosciences Salivary Cortisol ELISA (enzyme-linked immunosorbent assay) kit (cat. DCM020-9) from DiaMetra (Perugia, Italy). All samples were analyzed in the same trill—the assay sensibility was 0.8 nmol/L, and the inter- and intraassay variation coefficients were all below 10%, according to the manufacturer’s specification. In our laboratory, intra-assay precision was 2.3% and 2.5% and the inter-assay reproducibility was 3.2%.

### 2.4. Psychological Tests

The State-Trait Anxiety Inventory (STAI) is a validated and widely used instrument to measure anxiety level. In the current study, the Polish adaptation of STAI-state version was performed to determine the participant’s momentary (STAI X1) anxiety level. The overall (total) score for each part ranges from a minimum of 20 to a maximum of 80. Higher scores indicate higher values of anxiety in both subscales [23]. 

To evaluate stress management styles, the Polish adaptation of Coping in Stressful Situations (CISS) Questionnaire was used [23], which describes the level of task-oriented coping, emotion-oriented coping, and avoidance-oriented coping. Results are presented on three scales:

- TOC—task-oriented coping, which reflects undertaking attempts to manage the stressful situation, including cognitive reappraisal and information searching.
- EOC—emotion-oriented coping, manifesting as concentration on emotional experiences, and reducing emotional strain.
- AOC—avoidance-oriented coping, which can take two forms: engagement in alternative activities or social contacts to avoid confrontation with the source of stress.

The scores that can be obtained within each of the three scales range from 16 to 80. Higher scores represent higher levels of the measured strategy [23]. 

The International Physical Activity Questionnaire (IPAQ) was used to measure the intensity of physical activity in both groups. To calculate the weekly physical activity in energy expenditure units (metabolic equivalent of task per minute—MET/minute), the number of hours dedicated to each activity class was multiplied by the specific MET score for that activity [24].

### 2.5. Study Design

**Figure 1:**
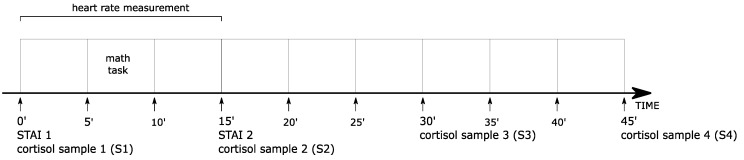
Graphic presentation of the experimental procedure.

First, interested participants were screened through a telephone interview to check if they met general eligibility for the study. Eligible participants then went through a detailed interview in the laboratory at which time they were informed on how to prepare for the study. Participants were instructed to arrive at the laboratory at least 3 h after awakening (to avoid the confounding influence of the cortisol awakening response). The experimental sessions took approximately 45 min to complete and began between 9 and 11 a.m. (as participants reported their wake-up time was usually between 6 and 8 a.m.). This time of the day was chosen to cause minimal interference with the athletic group’s training cycle. To minimalize the effect of potential factors influencing the HPA axis, participants were instructed to avoid mental and physical effort, sexual activity, and sleep disturbances (i.e., sleeping longer or shorter than usual, waking up during the night or napping during the day) within 24 h prior to the study. They were also instructed to refrain from alcohol during the 48 h prior to the laboratory session and abstain from caffeine intake in the 4 h prior to the study. Additionally, they were advised to drink only water and to not eat, brush or floss teeth 1 h prior to the session (as food particles in the saliva or trace amounts of blood that may result from teeth brushing can affect the hormone level). Figure 1 illustrates the study design. During the start of each study, participants were screened to check if they were following the instructions. If they were not (e.g., an unexpected stressful situation had occurred within 24 h prior to the laboratory session) or if a stressful event was expected within one week (e.g., exam, job interview, or final match), the participant was asked to come another day still within the follicular phase of her menstrual cycle.

Immediately before the task (at 5 min), each participant was informed that her performance was going to be voice recorded and reviewed by a panel of specialists. Then, the first saliva sample for cortisol (S1) was taken and state anxiety (STAI 1) assessed. The arithmetic task began after a 5-minute pre-task HR measurement. The serial subtraction task utilized in the experiment consisted of five 1-minute blocks of mentally (with no visual or paper clues) subtracting by 7s and 13s from a 4-digit starting number. However, no time pressure and/or feedback that could have induced a social-evaluative threat was performed [25]. After 5 min of recovery following the task (which reflects the recommended 15 min of anticipation [26]), the second saliva sample (S2) was collected and state anxiety (STAI 2) was assessed once more. Finally, subjects had 30 min to recover while they answered the CISS questionnaire. During this recovery period, saliva samples (S3 and S4) were collected every 15 min following termination of the task. 

### 2.6. Statistical Analysis

To investigate differences between groups regarding height, weight, BMI, age, menstrual cycle length, years of education, physical activity, stress coping strategies, and trait anxiety levels (volleyball players vs. non-athletes), a non-parametric Mann–Whitney U test or a parametric *t*-test was performed depending on data distribution. To investigate the stress response, a two-way ANOVA with repeated measures was performed with training status as a between-subject factor and time (for state anxiety, pre-task and post-task; for HR: before arithmetic task, arithmetic task, and following arithmetic task, and for cortisol: S1, S2, S3, and S4) as a within-subject factor. Salivary cortisol and state anxiety data which did not show normal distributions were transformed using Box-Cox-power transformation. At the core of the Box-Cox transformation is an exponent, lambda (λ). All values of λ are considered and the optimal value for data is selected; the “optimal value” is the one which results in the best approximation of a normal distribution curve [27]. Our results indicated that the optimal λ value was 0.54 (confidence interval: 0.26–0.78). However, the presented process works better if this value is rounded; this makes it easier to transform the data back and forth. Thus, we chose a λ value of 0.5, which falls within the confidence interval and represents the square root transformation. Furthermore, all participants were qualified as cortisol responders and cortisol non-responders, according to Miller et al. who classified individuals who show 15.5% increase in cortisol levels following the stress (which was, in our study, anticipated stress only) as responders [28]. Furthermore, responders’ cortisol levels were compared using a two-way ANOVA with repeated measures with training status as a between-subject factor and time (S1, S2, S3, and S4) as a within-subject factor. The Greenhouse–Geisser procedure was used when the requirement of sphericity was violated. Post-hoc planned comparisons were performed using Bonferroni adjustments for *p* values <0.05. STATISTICA 13.1 (Satsoft, Cracow, Poland) was used for statistical analysis. The level of significance was marked at *p* < 0.05. 

## 3. Results

### 3.1. Group Characteristics

There were no significant differences between volleyball players and sedentary women for age, weight, BMI, educational level (years of education), or menstrual cycle length (all *p* > 0.05). Volleyball players were, however, taller (*p* = 0.034) and showed greater physical activity (*p* < 0.001) compared to sedentary women (see Table 1). Trait anxiety level and coping strategies did not differentiate volleyball players and sedentary women.

### 3.2. State Anxiety

The repeated measures ANOVA showed the main effects on time (F_(1,60)_ = 62.00, *p* < 0.001, η^2^ = 0.51), and post-hoc analyses showed a significant increase in state anxiety in both groups (all *p* < 0.001), but no time × group interaction—F_(1,60)_ = 2.59, *p* = 0.11, η^2^ = 0.51.

### 3.3. Heart Rate

**Figure 2:**
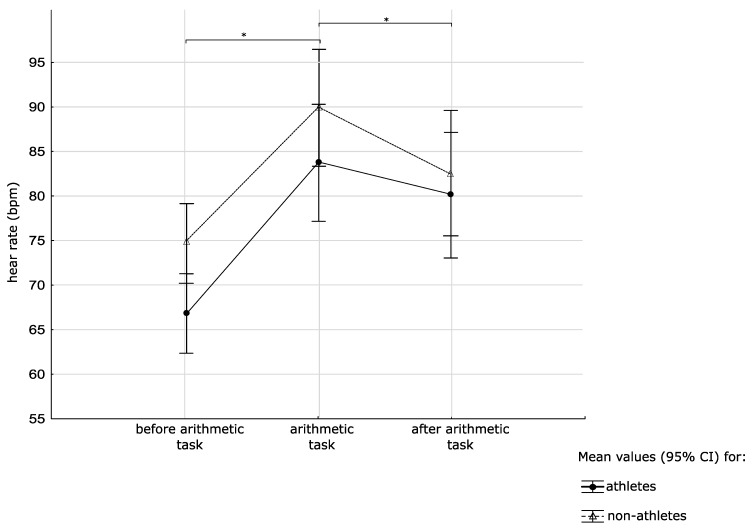
Mean values ± 95% confidence interval (CI) of response to the arithmetic task in heart rate in both groups (* significant differences between time points within a group).

To gain future insight into heart rate response to stress, repeated measures ANCOVA (time × training status) was performed. Significant effects of time (F_(2,120)_ = 21.34, *p* < 0.001, η^2^ = 0.26) were found, but not for training status (F _(1,60)_ = 2.69, *p* = 0.106, η^2^ = 0.04). Post-hoc analyses showed higher HR during and following exposure to the arithmetic task than before the arithmetic task (all *p* < 0.001) in both groups (see Figure 2).

### 3.4. Salivary Cortisol

**Figure 3:**
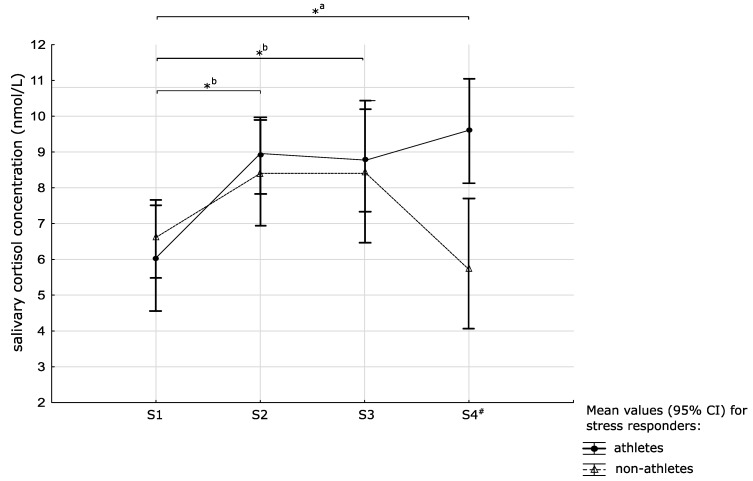
Mean values ± 95% confidence interval (CI) of response to the arithmetic task in salivary cortisol concentrations in both groups (* significant differences between time points within a group; # significant differences between volleyball players and sedentary women at the same time points).

The repeated measures ANOVA showed the main effects of time (F_(3,180)_ = 11.73, *p* < 0.001; η^2^ = 0.16) and training status (F_(1,60)_ = 4.69, *p* = 0.034, η^2^ = 0.07) and a significant interaction between training status and time (F_(3,180)_ = 3.07, *p* = 0.029, η^2^ = 0.05). Post-hoc analyses showed higher cortisol concentrations among volleyball players in S2 (*p* < 0.001), as well as higher cortisol concentrations in S2, S3, and S4 compared to S1 in volleyball players (all *p* < 0.001) and no significant differences in cortisol concentrations between S1, S2, S3, and S4 samples in sedentary women (see Figure 3).

**Figure 4:**
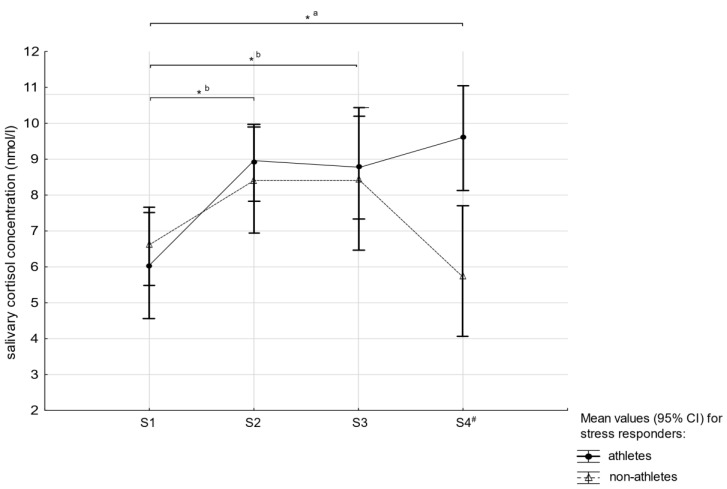
Mean values ± 95% confidence interval (CI) of response to the arithmetic task in salivary cortisol concentrations in both groups (*b: significant differences between time points within group of volleyball players and within group of sedentary women; *a: significant differences between time points within group of volleyball players; # significant differences between volleyball players and sedentary women at the same time points).

Furthermore, in each group, we identified cortisol-responders defined as individuals who showed a 15.5% increase in cortisol levels in S2 compared to S1 [28]. Hence, 21 volleyball players (84%) and 11sedentary women (37%) were classified as cortisol-responders. The repeated measures ANOVA showed the main effects of time (F_(3,90)_ = 5.66, *p* = 0.013, η^2^ = 0.16) and training status (F_(1,30)_ = 3.76, *p* = 0.041, η^2^ = 0.11), as well as significant interaction between training status and time (F_(3,90)_ = 3.24, *p* = 0.024, η^2^ = 0.10). Post-hoc analyses showed higher cortisol concentrations among volleyball players S4 (*p* = 0.011), higher cortisol concentrations in S2, S3, and S4 compared to S1 in volleyball players (all *p* < 0.001), and higher cortisol concentrations in S2 and S3 compared to S1 and S4 in sedentary women (all *p* < 0.05) (see Figure 4).

## 4. Discussion

During previous research, we observed higher HR during the “stress” (arithmetic task) condition [29], regardless of sports performance. Our study did not reveal differences in the autonomic nervous system (ANS) stress reaction between volleyball players and sedentary women; however, we observed a constantly lower, although not statistically significant, heart rate in volleyball players (*p* varying from 0.0531 to 0.0624, depending on time point). As previously observed, it is noteworthy that psychological and physiological attributes may vary greatly between female volleyball players [30], thus the lack of significant statistical differences may be due to the large variation of HR, particularly in the athletic group. Nikolaidis et al. proved that the maturity of volleyball players should also be examined when analyzing HR [31]. There was the possibility of variation of maturity within the group, as the group consisted of young females (approximately 25% of participants were in the age range of 18–20). 

Our results are in line with recent data, stating that training remodels the heart and reduces resting heart rate by altering the number of ion channels, which are key pacemakers which help to determine heart rate [32]. In this manner, the sinus node has an intrinsic ability to react against chronically increased heart rate in athletes and therefore lowers heart rate in highly trained individuals [26]. 

We also observed higher HR for both groups while in the “stress” (arithmetic task) condition. Our study did not reveal differences in ANS stress reaction between volleyball players and sedentary women. The cross-stressor-adaptation (CSA) hypothesis implies a stress-buffering effect at a good fitness level, which may lead to the assumption that top-level volleyball players, who are definitely at a good fitness level, should present attenuated HR response to an anticipated stressor. It is worthy to note that volleyball is not considered to be a strictly aerobic sports discipline, and that this type of sport has been shown to induce the best adaptation to other stressors [16,33]. Furthermore, the CSA hypothesis is based on physical fitness per se and does not include the fact that top-level sports performance, and in particular team sports, may be associated with many other factors, such as psychosocial stressors, that influence human performance [20,34]. These facts, together with the previously mentioned high variation in HR among volleyball players, may result in the lack of differences in HR response between groups. 

Interestingly, we observed a significant difference in cortisol response to the mock stressful situation between these two groups. The inconsistency of this reaction with HR reactivity may be explained by the flexibility of the cardiovascular system, manifesting in an easy return to initial HR values after a physically and/or psychologically stressful event [35]. Indeed, in their recent study, Wunsch et al. proved that the effects of stress on salivary ANS and HPA markers do not seem to be related [36]. Both the ANS and the HPA axis are distinct physiological systems that are involved in the human response to stress, although there are some interrelations between these systems. Our study also confirms the statement that these two systems seem to react independently. 

As the experiments were performed in the mornings, and no afternoon cortisol samples were collected, the small possibility of the potential role of increased before-noon cortisol concentrations on our results cannot be completely excluded. However, participants were instructed to arrive at the laboratory at least 3 h after awakening, and both groups did not differ in the time after awakening. Thus, the potential influence of elevated cortisol levels before task on altered cortisol response should be independent of the group. As only sedentary individuals presented altered cortisol response, it may be assumed that the time of measurement had a marginal effect on the results.

Compared to the levels in the before-session condition, higher levels of state anxiety were reported by the participants following the arithmetic task. Thus, all participants (both volleyball players and sedentary individuals) believed that the arranged situation was really intended to induce stress which mobilized them to do their best [37]. It is worth noting that, contrary to sedentary individuals, top-level female volleyball players may perceive psychophysiological testing of stress management and “getting greater scores” as more important to their future career, as it is obvious that coping well with stress is crucial for athletic success [9,26]. Interestingly, when comparing cortisol responders only, we found that only volleyball players presented prolonged cortisol response. 

However, while earlier studies have suggested that athletes have a lower cortisol response to stressors, newer studies sometimes show the opposite effect, which was presented in the recent meta-analysis [16]. This confusing inconsistency may result from methodological differences (i.e., different types of sports performance or regarding physical fitness per se). Our study involved top-level volleyball players, which are not only engaged in regular physical activity but are also under great pressure as they are fully paid for their sports performance. Thus, dealing successfully with competitive and organizational issues is essential to providing their livelihoods. That is why volleyball players may perceive an anticipated stressor as a greater challenge than sedentary individuals.

In response to a situation perceived as a challenge, cortisol is released to increase arousal and attention and suppress pain, and thus help the body successfully deal with stressors [38]. The role played by cortisol is vital to physical as well as mental performance because of its catabolic, proteolytic, and anti-inflammatory characteristics and its functions to maintain blood pressure and plasma volume. In our study, volleyball players showed higher salivary cortisol concentrations following the arithmetic task. The behavioral pattern for the population who performed several bouts of intense exercise each week makes it difficult to evaluate the different sources of cortisol. According to data from Luger et al., repetitive high physical activity leads to alterations in cortisol levels [39]. These findings are in line with studies showing that acute bouts of vigorous exercise are associated with a considerable increase in salivary and plasma cortisol concentrations [33,40,41]. However, in the current study, neither group engaged in physical activity at least 24 h prior to the experiment. 

The Theory of Challenge and Threat States in Athletes (TCTSA) is a model explaining how athletes react psycho-physiologically within competitive situations [42]. In general, a positive perception of anxiety symptoms and stressful situations as a challenge is reported by elite performers in comparison with non-elite performers [43] and is associated with higher performance levels [42,44,45]. This may explain elevated cortisol levels in response to anticipated stress in preparation to manage the expected challenge—in particular, physical performance [43]—even if this is not necessary. As previous studies have shown, the HPA axis is not particularly sensitive to mental arithmetic tasks [46], and it may be assumed that the anticipation of challenging events elevates cortisol levels in volleyball players. 

As the peak in salivary cortisol is usually observed 10–30 min after stress cessation, it can be concluded that cortisol levels continued to elevate after the “stress” condition in female volleyball players. Maintaining elevated cortisol levels could then be a kind of physiological adaptation to stressful situations among volleyball players even if these athletes may not have been aware of their physiological reaction to an anticipated stressor and therefore report similar anxiety levels to controls [29,38,43,47,48]. On the other hand, the observed mechanism may be maladaptive, and reduce the athlete’s performance when challenging situations appear too often. Earlier studies have revealed significant relationships between salivary cortisol levels and lower performance scores and higher fatigue [49]. Those results are not surprising as cortisol can inhibit protein synthesis and thus lead to decreased muscle mass by its catabolic action and muscle strength and power connected with the improved athletic performance [50]. 

Cortisol concentrations following stressful situations can also be affected, at least in part, by an individual possessing more efficient cognitive strategies for coping with stressful situations [40]. However, the current study revealed no differences in coping strategies between volleyball players and sedentary women. Given that cortisol secretion has been suggested to be sensitive to differences in task design [26], caution is suggested when comparing our findings with previous literature. It can be assumed that our results could be explained by (1) intense athletic performance and physical fitness per se and (2) stress anticipation, as the fact of entering the study may have been perceived as a type of competition by volleyball players but not by the sedentary women. These explanations can also remain independent or be complementary.

### 4.1. Strengths and Limitations of the Study

The size and homogeneity of the present study, as well as the artificial conditions of the task, limit the ability to generalize our research findings. As the experiments were performed in the mornings, and no afternoon cortisol samples were collected, the small possibility of the potential role of increased cortisol concentrations in volleyball players cannot be completely excluded, specifically regarding this time of the day. The current study did not involve any non-athletes who performed regular physical activity; therefore, it is indistinct whether the changes observed were due to a high level of physical activity per se or to top-level sports performance. 

On the other hand, subtle changes in cortisol release in response to stress can be hindered in a non-homogenous sample. This study was designed to reduce possible confounders to a minimum, in particular reducing those connected with female sex hormones to a minimum through restrictive exclusion criteria. 

### 4.2. Practical Applications

The current study highlights the effect of an anticipated stressor in prolonged cortisol release among female volleyball players. Over the long term, frequent boosts in cortisol levels may lead to a decrease in muscle strength as well as physical and mental fatigue and, thus, a reduced athletic performance. Therefore, coaches should control the frequency of challenges posed to volleyball players to prevent unnecessary cortisol stimulation.

## 5. Conclusions

It may be concluded that female elite volleyball players seem to differ in cortisol response but not cardiovascular and subjective (psychological) graduation of stressors when compared to sedentary-lifestyle individuals, at least in the mornings. This may perhaps relate to athletes’ physiological habituation to stressors, as exposure to training-based stressors seems to promote stress-related response that can be observed prior to any anticipated, non-specific stressors.

## Figures and Tables

**Table 1:** Comparison of age, height, weight, body mass index (BMI), menstrual cycle length (days), years of education, physical activity (MET × min/week), and psychometric variables (state anxiety and coping strategies) between volleyball players and sedentary women.

Variable	Athletes			Non-Athletes			*p*
	*n* = 25			*n* = 30			
	Me	Q1	Q3	Me	Q1	Q3	
Age (years)	22.0	20.0	23.0	23.0	21.0	24.0	0.200
Height	177.3	172.1	180.1	173.1	169.8	175.9	0.034
Weight	73.5	68.5	76.1	71.2	67.4	74.9	0.081
BMI (kg/m^2^)	23.5	22.25	24.76	24.44	23.24	25.14	0.060
Menstrual cycle length (days)	28.0	27.0	29.0	28.0	28.0	29.0	0.143
Years of education	14.0	13.0	14.0	14.0	14.0	16.0	0.055
Time from awakening (min)	192.0	154.0	210.0	175.0	155.0	213.0	0.888
Physical activity (MET min/week)	2700.0	2250.0	3700.0	420.0	340.0	550.0	<0.001
Psychometric variables:							
State anxiety (points)	38.0	36.0	39.0	38.0	36.0	40.0	0.994
Coping strategies							
Task-oriented coping (points)	59.0	57.0	62.0	60.0	55.0	63.0	0.927
Emotion-oriented coping (points)	46.0	41.0	54.0	47.0	40.0	59.0	0.668
Avoidance-oriented coping (points)	36.0	32.0	41.0	39.0	28.0	44.0	0.778

Me: median; Q1: lower quartile; Q3: upper quartile, BMI: body mass index; MET: metabolic equivalent of task.
